# Did Human Microbes Affect Tourist Arrivals before the COVID-19 Shock? Pre-Effect Forecasting Model for Slovenia

**DOI:** 10.3390/ijerph192013482

**Published:** 2022-10-18

**Authors:** Sergej Gričar, Štefan Bojnec

**Affiliations:** 1 Faculty of Business and Management Sciences, University of Novo Mesto, Na Loko 2, 8000 Novo Mesto, Slovenia; 2 Faculty of Management, University of Primorska, Izolska Vrata 2, 6000 Koper, Slovenia

**Keywords:** autoregressive integrated moving average model, forecasting demand, microbial infections, pandemic, shocks in tourism and public health

## Abstract

In 2020, with a substantial decline in tourist arrivals slightly before the time of COVID-19, the innovative econometric approach predicted possible responses between the spread of human microbes (bacteria/viruses) and tourist arrivals. The article developed a conceptually tested econometric model for predicting an exogenous shock on tourist arrivals driven by the spread of disease using a time series approach. The reworked study is based on an autoregressive integrated moving average (ARIMA) model to avoid spurious results. The periods of robust empirical study were obtained from the data vectors i) from January 2008 to December 2018 and ii) from January 2008 to December 2020. The data were obtained from the National Institute of Public Health (NIPH) and the Statistical Office of the Republic of Slovenia. The ARIMA model predicted the number of declines in tourist arrivals for the approaching periods due to the spread of viruses. Before the outbreak of COVID-19, pre-pandemic results confirmed a one-fifth drop in tourist arrivals in the medium term. In the short term, the decline could be more than three-quarters. A further shock can be caused by forecasted bacterial infections; less likely to reduce tourist demand in the long term. The results can improve the evidence for public health demand in risk reduction for tourists as possible patients. The data from the NIPH are crucial for monitoring public health and tourism management as a base for predictions of unknown events.

## 1. Introduction

External factors such as crises disrupting tourism demand can substantially influence tourist arrivals and related public health management in tourist destinations. From the conventional econometric demand theory, tourist arrival is a dependent variable. On the other hand, the independent variable has now changed from the traditional ones (prices and income) to extraordinary ones (microbes) that were not previously (before the year 2020) researched for prediction purposes [1]. Such an approach is essential in econometrics to discover new pre-effect shocks. Therefore, the motivation of the study is to present the validated pre-effect study of threatened microbes to a tourist arrivals demand theory [1].

Notwithstanding, before the COVID-19 pandemic, some authors [2] warned of a decline in tourist arrivals. This was due to the possible significant spread of viruses. In contrast to the steady growth of tourist arrivals, the study predicted possible non-linear developments with health shocks and rapid declines in tourist arrivals. The 2020 pandemic was not the first crisis that broke out [3]. However, the global health crisis and the rapid decline in tourist arrivals, with negative economic implications, were unexpected for the vast majority of tourism managers and health institutions.

This article contributes to the analysis of cyclicity in tourist arrivals associated with the spread of viruses, where a drop in tourist arrivals for the tourism economy can have a sudden and unexpected effect. An apparent novelty and contribution are twofold. The first is a literature review on predicting microbe threats of pre-pandemic (viruses) and post-pandemic (bacteria) shock. The second developed and widened the prediction models for tourist arrivals in a receiving country in association with human microbes health data that can have implications for public health in tourist destinations. A common thread running through the article is the tourist as a patient, first analysed by Sorokin and Payda in 1975 [4].

The primary purpose of this paper is to rebound the importance of forecasting future shocks before they appear. Additionally, the validity of time series is tested for a broad range of sciences in economics and health sciences. Overall, this article presents the predicted shock of viruses which was forecasted slightly before the pandemic occurred. Henceforth, disseminating such time series forecasting is essential to accurately predict future shocks like tourism expansions. Performing this, the secondary data and non-traditional variables choice is a goal of this state-of-the-art research. Therefore, the following research hypothesis is tested:

**H1.**  *Tourist arrivals would sharply decline due to virus epidemics (viral infections) but not due to bacterial infections. Both types of diseases are caused by microbes and can be spread by infected people*.

The opposite alternative hypothesis is:

**H1_0_.**  *Tourist arrivals are an immune factor to external shocks like microbes*.

Hypothesis testing follows some of the most recent approaches to this theme. Their results are presented in Appendix A. The most critical finding and conclusion are regarding the role of international tourism as a possible factor in the outbreak and early spread of diseases worldwide. Given the information, health shocks are expected to become more frequent.

The article is organised as follows. The next section presents the literature review. Further units present the methodology, data, and empirical results with discussion and implications. The final section concludes.

## 2. Overview of Some Shocks to Public Health and Tourist Arrivals

Some authors [3,5] reported that tourism suffers from several crises, the most obvious being the economic crisis of 2007/2009. Among others, some interruptions in tourist arrivals were driven by the severe acute respiratory syndrome [SARS] outbreak in 2003 and the volcanic eruptions in Iceland in 2010 and 2014. The year 2019 was marked by an overwhelming increase in tourist arrivals [6] but since then has been a substantial decline caused by COVID-19 [7], which has motivated our research to present the pre-pandemic projections which have predicted the volatility in tourist arrivals confronted by viruses using time series data. Therefore, our study focuses on predicting tourist arrivals in association with microbes.

This article aims to develop a testable pre-pandemic model to predict the proliferation of viral diseases [8]. Following the previous study [2], we aim to forecast the association between tourist arrivals, bacteria, and viruses (what the data tell us when speaking freely). It is expected that there are inverse relations between them. The future developments can be predictable but recently less precise, which has stipulated this study to develop a testable model for predicting the effects of the microbes on tourist arrivals.

Despite the importance of tourism in national economies and the high transmission of diseases, a sudden chain reaction affects tourism growth [9,10]. The effects of stochastic terms and possible post-pandemic detection of these external shocks on tourism are widely recognized [11,12]. 

Overall, one of the threats to tourism growth is microbes [13]. Some authors [14] identified food safety and health information as important destination reputations. Until the pandemic, when the crisis started at the end of 2019 and early 2020, there was rare research about a decline in tourist arrivals due to microbes and their associated costs to society [2,5,15], tourism, and destination management [16,17].

### 2.1. Modelling and Literature Overview

Following [18], the primary purpose of the current research is to present the results of an econometric approach for a prediction of an “unexpected” shock in the health of people to analyse an exogenous event [19]. The researchers assume that each tourist could also be a (potential) patient. This requires establishing infrastructure and networks between the tourism economy and medical centres to delineate where tourists can find medical aid [20]. Therefore, the main objective of the research is to bridge the gap in tourism research literature between the following:

1. Post-effect research (analysing already known events);
2. Pre-effect research (detecting and discovering prophecy).

Some authors [5] recognise these elements as the defining basis for the occurrence infection phase. While only a few tourism and public health researchers use predictive statistical methods [8], most research reports have known and defined problems. Some articles on the COVID-19 pandemic [21,22,23,24,25] confirmed the gap in the literature on a lack of research on predicting the outbreak of the viral disease based on virus spread using a quantitative methodology [8] with underdeveloped predictive power [26]. Based on a systematic review of the literature, the missing research on predictive tourism crises was reported [5]. On the other hand, based on time series data, some authors [24] predicted a massive post-effect research decline in tourism during the pandemic. The studies of past events in predicting future paradigms on reliable modelling have been limited [27] and the most relevant articles have been published after 2019. They can be selected based on the search keywords predicting, forecasting, prognoses viral, bacterial, or microbial outcomes in tourism. The collected data and methods applied are essential for predictions of future performance [26].

The econometric model with the specification of variables plays a vital role in predicting future events, shocks, structural break disruptions in tourism, public health and time series volatilities [6,11,26,28,29]. The idea of the perfect econometric method was developed by [16], who identified the autoregressive integrated moving average [ARIMA] as a suitable model for detecting infectious disease outbreaks. In addition, authors [30] use this method to predict some other infections and [31] recommend applying a non-linear ARIMA model. The volatilities in time series and their implications have been investigated in different tourism-linked activities such as financial [32], banking [33], and cultural [34] institutions. They applied an econometric approach.

### 2.2. Empirical Phenomena of Pandemic Pattern and Public Health

There is a growing interest in tourist motivations for visits to the gastronomy of different cultures with a food neophobia [35]. Travels in the global economy require transportation, overnight, and food and beverage service providers on the market [36]. Foodborne or food-related infections represent a significant and growing public health and economic problem in many countries. Although food-related infections, poisoning, and illnesses can be severe and fatal, mild cases with regular monitoring were often undetectable or underestimated. The high incidence of diseases can have high costs to society, both material (financial) and intangible (disability, pain) [6]. Some authors [37] discuss the leisure vs. health nexus and confirm synergies between the concepts.

Any person travelling as a tourist who does not have a specific health defect or illness would impede mobility. During their trips, tourists need services, food, and water for basic physiological human needs, which can be exposed to food and water poisonings [38]. Moreover, air-conditioning for casual well-being could dress bacterial and viral infections. To spread infections, tourism and social and sexual activities are a perfect match for spreading diseases in the short-term or long-term [39,40].

We focus on Slovenia, the Central European country at the intersection of the Alps and the Adriatic Sea. Slovenia is a small open economy in Central Europe, with Ljubljana as the capitol city. Slovenia has been a member of the European Union since 2004. Slovenia’s neighbours are Austria to the north, Italy to the west, Croatia to the south and Hungary to the east. See Appendix C for the geo map of Slovenia.

The analysis aims to reduce and manage a shock in tourist arrivals from a gigantic viral infection, consequently, COVID-19. In early 2021, Eurostat [41] published the statistical data where Slovenia had 33% more domestic tourist arrivals than a year earlier (e.g., 2019) and an overall decrease in tourist arrivals (domestic and foreign) of about 40%.

## 3. Methodology

The ARIMA method was applied to test our hypothesis, with tourist arrivals as the dependent variable following Wong et al. [42]. In econometrics, especially in time series analysis, the ARIMA model generalises the autoregressive moving average (ARMA) model. ARIMA and ARMA models are fitted to time series data to understand the data better or predict future points in the series (forecasting). ARIMA models are used in some cases where the data show evidence of non-stationarity in terms of mean, where an initial differencing step can be used more times to remove the non-stationarity of the mean function.

In the ARIMA (p, d, q) (P, D, Q) model, the symbols mean the following: p/P is autocorrelation; d/D is integration, and q/Q is moving average. The capital letters (P, −D, −Q) denote seasonal entries, and the lowercase letters (p, d, q) represent non-seasonal attributes. The ARIMA model is recognised as a suitable econometric approach for economic predictions and the forecasting of tourist arrivals. The ARIMA model has a constant; the regression coefficients show the direction and dimension of the relationship between the dependent variable $y_{t}$ and the one-period lagged independent $x_{t-1\;}$ variables. 

This research uses time series data and the relevant offices collected secondary data. In modern econometrics of time series, it is evident that approaching past events is essential to predict future events. Moreover, it is necessary to spread the methodology to other sciences, to avoid collecting data by questionnaires or similar, while data has the most information on past events. It is known that in econometrics, past events generate future events with a significant length of observations which is more than 100. Nevertheless, the importance of econometric empirical testing is to choose relevant variables. A specific way to select the variable is regarding the methodology and problem which will be researched. Notwithstanding in time series is that the relationship between variables is calculated by significance and misspecification tests. This is important to define a reliable model. More specifically, the present research uses excellent ways to test, predict and disseminate pre-effect shock on time series by applying the ARIMA method.

**Figure 1:**
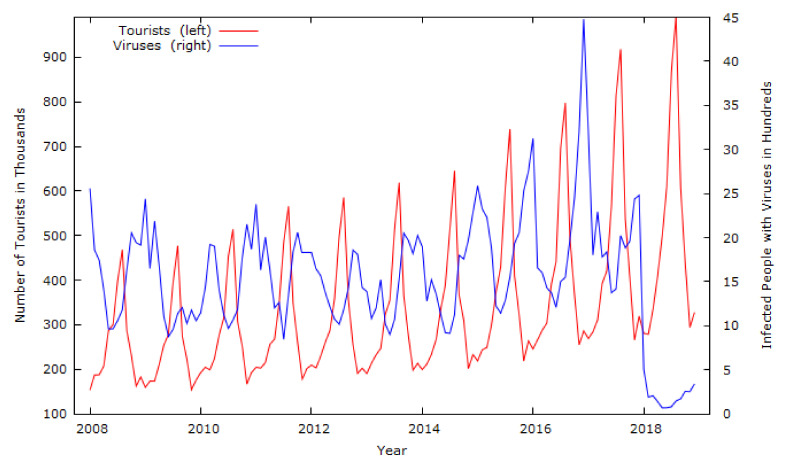
Data in levels for tourist arrivals and viruses from January 2008 to December 2018. Source: Authors calculations [43,44].

The secondary data were obtained from the National Institute of Public Health (NIPH) of the Republic of Slovenia [43] and the Statistical Office of the Republic of Slovenia’s (SORS) SISTAT website [44]. The data on 12 prevailing infections in Slovenia were obtained from the NIPH [44]. The data on tourist arrivals for the same periods were obtained from the SORS [43]. Tourist arrivals are a dependent variable and are accompanied by viruses, as shown in Figure 1. 

Additionally, testing the seasonal patterns of tourist arrivals and microbe influences is introduced by the dummy variable (Appendix B). It is equal to 1 for November to April (low season, winter months) and 0 otherwise for the rest of the year, representing the higher season and summer months. Tourist arrival is a time series variable that measures the number of specific tourist arrivals in a country. 

A domestic tourist is a person with a permanent residence in Slovenia who temporarily resides in another place in Slovenia for leisure, relaxation, business, or other reasons and who spends the night at least once (at least one night) in a restaurant or other accommodation establishment in this place. A foreign tourist comes from abroad and settles temporarily in an area in Slovenia for leisure, relaxation, business, or other reasons and who spends at least one night (at least one night) in a restaurant or other accommodation facility. The SORS determine the nationality of foreign tourists according to their country of residence. Whereby SORS start from the assumption that the country of residence is the same as the country of citizenship that was recorded when the tourist registered at the accommodation facility.

Variables of tourist arrivals, where April 2020 is set to number 1 from 0 due to the national lockdown, and the 12 most common microbes were tested and treated with a large temporal data series that consists of:

1. One hundred thirty-two (132) monthly observations from January 2008 to December 2018. This was an initial pre-effect period and the main goal of disseminating the research. This part is presented in the next section, the Results section;
2. One hundred fifty-six (156) monthly observations from January 2008 to December 2020. This is post-effect research and a subsequent goal of the investigation, which is presented in Appendix B to gain the robustness of the study. On top of that, the dependent variable was split into domestic tourist arrivals and foreign tourist arrivals. Foreign tourist arrival was tested separately for the country of origin, where the three most crucial outbound countries were used, e.g., Germany, Austria, and The Netherlands.

**Figure 2:**
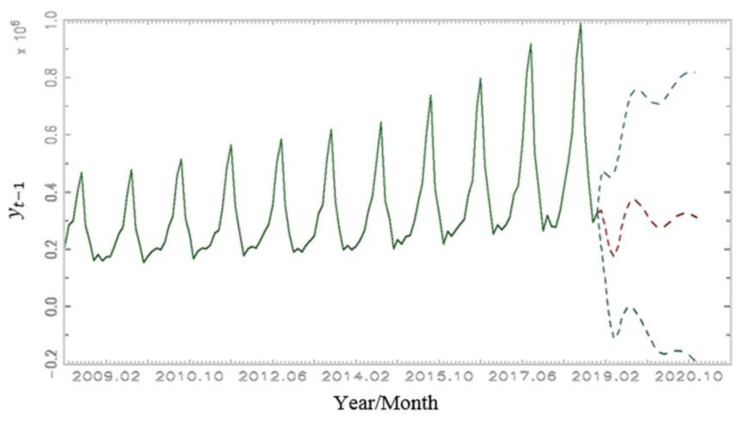
Forecast of tourist arrivals for 24 months. Source: Authors’ calculations. Note: Data vector is from January 2008 to December 2018; upper, middle, and lower forecast levels are from January 2019 to December 2020.

The vector error correction model (VECM) is introduced to mark up the plotting of predicting events. The VECM is an applied methodology that boosts the ARIMA modelling in more advanced testing (Figure 2).

## 4. Results

The 12 most common infections were included in the ARIMA (p,−d,−q) model as independent variables to test *H1*. After containing 132 monthly observations for all 12 independent variables in the ARIMA model, the reference model examined regressors that most illustrate the association of viral disease infections on the dependent variable (tourist arrivals):

(1) $$tourist\;arrivals_{t}=1103.00+3807.00\cdot E.Coli_{\begin{array}{c} t-1\\ \left(0.01\right) \end{array}}+21,061.00\cdot campylobacter_{\begin{array}{c} t-1\\ \left(0.00\right) \end{array}}-41.81\cdot Viruses_{\begin{array}{c} t-1\\ \left(0.01\right) \end{array}}+\varepsilon_{i\;j}$$

where p values are in parentheses.

The value of the adjusted deterministic coefficient is 0.23. The stable ARIMA model of one-lagged autocorrelation, first integration, and the first order of moving average in a non-seasonal part of the model (1,−1,−1) and an omitted seasonal part (0,−0,−0) explains 23% of the variability in tourist arrivals in Slovenia with the specified bacterial and/or viral infections. Therefore, the trending ARIMA model is $\left(1,1,1\right)\cdot {\left(0,0,0\right)}_{12}$, where the number 12 indicates the autocorrelation on the twelvefold lag, which is the recognisable definition for the monthly time series.

The results suggest that tourist arrivals in Slovenia were not threatened by the identified infections of *E. coli* and Campylobacter. Despite the recognised presence of these two bacterial infections in the country, tourist arrivals increased.

The main threats to sustainability in tourism arrivals were virus infections and the spread of diseases [8]. Our empirical result reveals that each case of illness was linked with a reduction in tourist arrivals by 41.81 persons per month. 

In 2020, during the epidemic surge, the decline in tourist arrivals was estimated at 75.43%. The data vector is compiled from 1 February to 24 December 2020; the number of tourist arrivals is 2,762,394 and the number of COVID-19 patients is 112,395, while in 2019 the number of tourist arrivals was 6,229,573 [41,42,43,44,45].

When the ARIMA model contains the first differences in a seasonal part in its structure, the model becomes ARIMA (1,−0,−1) (0,−1,−0), where sepsis becomes the viral part of the connection for tourist arrivals.

Overall, the main results of this research show that pre-effect diagnosis can be crucial for different sciences. One can see that secondary data and reliable methodology could gain forecasting shocks. Therefore:

1. First, tourism declined on a large scale in 2020 [3,5];
2. Second, the decline was predicted on a pre-effect basis in 2019 [2];
3. Third, two non-conventional variables were treated in reliable modelling;
4. Fourth, the ARIMA model was used to forecast the event;
5. Fifth, a dummy variable for seasonal patterns of virus spread is added;
6. Finally, the tourism boom could be based on bacteria (*E. coli*, Campylobacter) in 2022/2023.

Furthermore, in Appendix B, the robustness of the results is tested. For country-specific cases, the results show the validation of the model and confirm that seasonality negatively affects tourist arrivals for both domestic and foreign tourists. On the other hand, bacteria have a positive effect on tourist arrivals. Nevertheless, viruses and COVID-19 have only limited prediction power for the next period and it is still negative but applicable only to Austrian, German, and domestic tourists. Notwithstanding, the overall effect of viruses (including COVID-19) on foreign tourist arrivals is not statistically significant for the next period measuring from the data vector 2008–2020, where 2008 is a basis year (2008 = 100) (Appendix B).

## 5. Delimitations and Limitations

### 5.1. Delimitations

Possible limitations of the study include testing the hypothesis for a single country using the non-seasonal ARIMA model. Therefore, other methodological approaches and public health data, such as a VECM and hospitalisations of tourists, should be considered. Figure 2 shows the reliability and credibility of such a VECM, which includes regressors from the presented ARIMA modelling. The VECM dramatically illustrates the massive decline in tourist arrivals in 2020 and after [Figure 1]. The predicted reduction in tourist arrivals (Figure 2) cannot be directly connected only to a COVID-19 outbreak which could be a consequence (symptom) and not a mitigate (muster, cause) problem for tourist arrivals.

### 5.2. Implications

**Figure 3:**
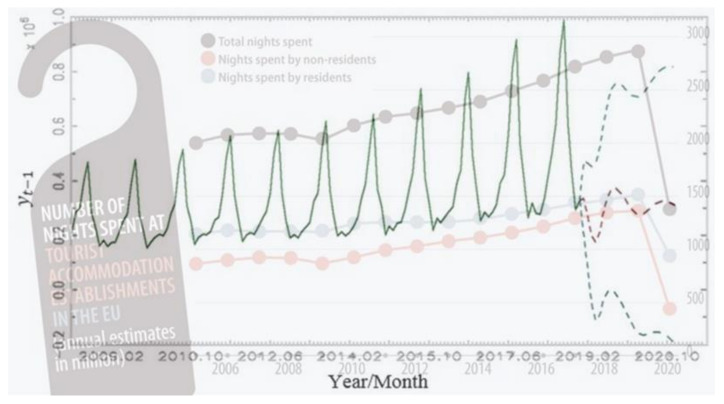
Forecast and realisation of tourist arrivals in overlay images. Source: Authors calculations, [47].

The result has immense implications for science. The predicted feature was overlaid with two images to confirm the modelling structure and reliable results (Figure 3). The first overlaid image is the Eurostat report on the decline in tourism demand in the European Union, and the second overlaid image is in Figure 2. It is obvious that the prediction of some authors [2] was incredibly accurate and the adapted result of this study significates the decline in tourist arrivals. Moreover, from the overlaid images (Figure 3), it is evident that the theoretical epidemic, i.e., the spread of the viruses, started in 2019 which was before the confirmation of the spread of the COVID-19 outbreak [46].

This is a novel contribution to science, first by conducting reliable modelling (pre-effect research), second by using the correct variables in the applied approach to public health (post-effect research), and finally by combining strategic intuition and specific field knowledge of the authors. This is the first attempt to disseminate knowledge about the impact of significant microbial influence on tourist arrivals. The surprising results were recognised before 2019/2020 as a pre-effect approach [2].

The study has broader policy implications for proper tourism management and public health adjustments to an unexpected event. All shocks can be predictable, including the recent pandemic [48]. According to Juselius [26], all events are predictable but only if researchers use the right and reliable methodology. This research provides evidence of the spread of microbes and their impact on tourist arrivals before the pandemic outbreak.

The implications for the management of patients are abundantly clear. The result clearly shows that any viral shock would lead to a decrease in tourist arrivals, while a bacterial impact would be less likely to decrease tourist arrivals. Therefore, one of the determinants of a post-pandemic tourism boom could be due to bacterial infections among tourists [49]. This is a two-sided precautionary effect, firstly to ensure the food safety of tourism employees and management, and secondly for the health sector to tender where tourists will be treated in case of infection. The latter is essential because of bacterial infections like *E. coli* and Campylobacter.

Second-line implications for society recognise possible future shocks in tourist arrivals due to viral infections and the emergence of diseases which will likely include HIV/AIDS and COVID-19. Both have been confirmed as significant future risks and threats to the tourism economy and public health, especially HIV/AIDS, which is recognised as the fourth researched threat in tourism [40,50,51,52].

### 5.3. Limitations and Discussion

This study shows that the three most common microbes explain 23% of the differentials in tourist arrivals. Other factors like price or income could explain the remaining 77% variance. Nevertheless, it is a widespread unconventional analysis that also includes other variables in econometric modelling, which has been performed in this research. The second limitation is that the testable model includes incoming tourists (export of services) and infections in a receiving country. The motivation behind this assumption is that each tourist is a potential patient. Therefore, we looked at the significance between the dependent variable (tourist arrivals) and the independent variables (microbes). Other drivers of infections for tourist arrivals in the outgoing country could be analysed. Finally, the monthly data could ignore some infections between the dates. 

Overall, the demand for tourism with tourist arrivals can be generated if tourists are not sick wherever they are, at home or already in a tourist destination. Therefore, this is a limitation of the study, but the significance of the model provides a reliable ARIMA process.

## 6. Conclusions

### 6.1. Theoretical Implications

The proposed research is based on econometric prediction modelling using time series. The study’s relevance is shown in the Figure in Appendix D, in which the United World Tourism Organisation (UNWTO) forecasts world tourism trends as linear and without shocks, which can be less realistic. The challenge is how to promptly predict a quantitative event, a surprise, and the period in which it can be expected. Accordingly, the main goal of the research was achieved, with the help of relevant data, to predict specific shocks in the future before they become a reality.

Synthesised, we explored how different forecasting models or modelling approaches, including time series and econometrics, can be applied to additional secondary data.

Therefore, the research contributes to the econometric model for predicting links between health events and tourist arrivals as crucial for economics, public health, and management sciences. As a novelty, this study spotlights and outlines the empirical attempt that has used a conventional time series methodology with the applied ARIMA and VECM models to predict shocks due to microbes. Therefore, this is the first attempt and research that confronts microbes and tourist arrivals using an approach based on secondary data properties. The predicted decline in tourist arrivals in 2020, supported by the presented econometric model due to the coronavirus disease (COVID-19) pandemic viral infection in Slovenia, is 75.43%. The result is confirmed by the latest report from Eurostat [41] with an overall decline in tourist arrivals, which is 40%, mainly by foreign tourists.

On the other hand, some bacterial infections cannot necessarily cause a decline in tourist arrivals. Despite their presence, there can even be an increase in tourist arrivals. The bacteria that were obtained and escalated in ARIMA are *E. coli* and Campylobacter. Besides the more publicly known bacterium Salmonella, which has no statistically significant effect on tourist arrivals in Slovenian tourism, both bacteria could be present in water and food. Their incidence is often limited to a particular hotel and restaurant or similar tourist dining and accommodation venues. Especially in private dining spaces like apartments, open fireplaces (BBQ), and Airbnb accommodations, the above bacteria could frequently spread, especially in warm weather [43].

### 6.2. Managerial Implications

The tourism industry should cooperate and network with the health system(s) and similar services that tourists can use. This can be supported by employee awareness and education improvements, accurate information, and designated first-aid spots for tourists (e.g., sign-posted meeting points). Therefore, the research results have a significant scientific, managerial, quality, policy, and practical implications relevant to tourism management: each tourist could be a potential patient. Thus, H1 is confirmed. 

Endmost, COVID-19 will likely be under control after four years of its appearance, while other threats to tourist demand will remain [48]. Lessons learned are in raising awareness of the importance of spotting the meeting points in health centres and disseminating the information on health prevention measures in accommodations and other tourist/guest facilities. Data from the NIPH are critical for monitoring public health and tourism management in predicting unknown events. Therefore, among future research issues is using the existing differentiated secondary health care data instead of the conduction of surveys and interviews widely used in public health research. 

To our best knowledge, none of the literature has shown signs of approaching the pandemic. Therefore, this article fil the gap to use historical data to analyse and discuss the possible risks in the future. These issues are of most importance for managers, policymakers, and practitioners in professional development in profit- and non-profit-oriented organisations. Moreover, non-government organisations could reach the goal of dissemination of such analyses. Therefore, this research adds some important insides to traditional tourism demand research [53,54,55] and country-specific research [56].

### 6.3. Future Research

The NIPH data for 2021 and 2022 are not yet available. As a result, the proposal for additional research in an ex-ante health crisis is a prediction that could introduce new viruses. The COVID-19 pandemic was not initially included in the study and pre-effect forecasting during the observed period (2008–2018). This was because a new virus had not yet spread throughout the population. However, as this article demonstrates, testing pre-effect predictions can be helpful for policymakers and practitioners. It makes use of historical data. On the other hand, the post-effect [56,57,58,59,60] projection can be applied to the most recent data period. This can serve as a foundation for future research processes, including sentiment analysis and data envelopment analysis [61].

Overall, country-specific research could be carried out, and the ARIMA model on inbound and outbound tourist arrivals is envisaged using the proposed model by incorporating the 12 most common microbes. As a result, combining the variables could provide an overview of disease spread among travellers, tourists, and other parts of the population.

### 6.4. Conclusions in Short

Our research, motivation, and relevance are essential to the tourism and health sectors. The usual drivers of demand and supply were constraints during the pandemic when each tourist could potentially be a patient. Therefore, the idea behind the study was to test whether the host country’s microbes significantly impact the spread of diseases among tourists using advanced econometric methods. Since April 2020, most Slovenian tourist destinations have had increased domestic tourist arrivals.

Therefore, the study has answered the question that microbes affected tourist arrivals before the COVID-19 pandemic. In other words, viruses have a negative and statistically significant effect on tourist arrivals in the forecasting period for 2019 and 2020. On the other hand, the subsequent period shows a positive and significant impact on *E. coli* and Campylobacter. Therefore, the initial research hypothesis cannot be rejected. Hitherto, the next period has positive vibes for tourism demand and public health.

Finally, the results’ reliability is examined across a broad time horizon up to 2020. The data on the COVID-19 virus has been added (Appendix B). To determine the significance of microbes on domestic and international tourist arrivals, a model is tested inside distinct country-specific tourist arrivals. 

The academic ramifications include: first, defining pre-effect forecasts using secondary data, and second, emphasising the usefulness of econometrics in the management, monitoring, and predictions of events relevant to health policy. To sum up:

- The first examined period (2008–2018) forecasted a decline in tourist arrivals due to viruses. Each infected person in the destination country (e.g., Slovenia) affects a decline in 41.81 tourists. On the other hand, *E. coli* and Campylobacter have opposite consequences on tourist arrivals. Each person infected by *E. coli* in the destination country (e.g., Slovenia) modified tourist arrivals by a rise of 3807 persons and by Campylobacter a rise of 21,061 travelers per month;
- The second studied period (2008–2020) confirms the robustness of the predicted results. This is presented in several ways. First, the overlayed images (Figure 3) show the expected and actual outcomes. Second, the wave in tourist arrivals in 2019/2020 using the ARIMA and VECM modelling procedure was exactly predicted, which validates the choice of variables and methods. The decline in 2020 was 75.43%. Finally, the expanded and split analysis is presented in Appendix B. These results confirm the forecasted wavering in tourist arrivals.

## Figures and Tables

**Figure A1:**
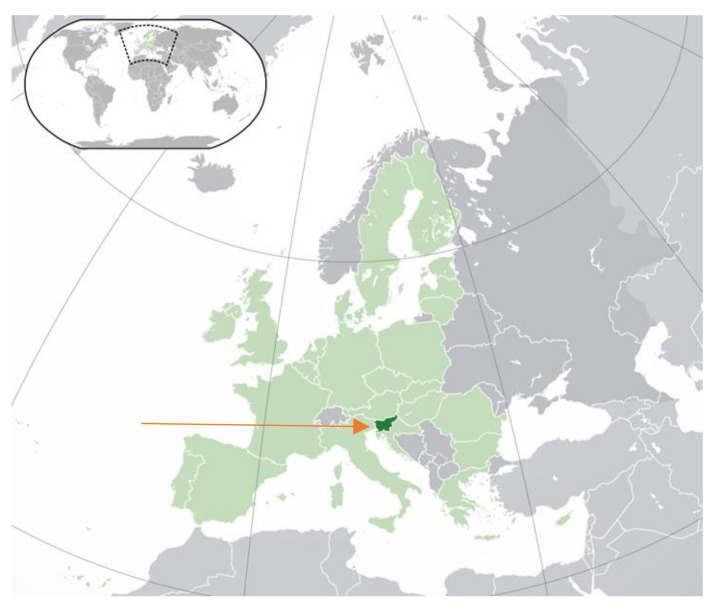
Geolocation of Slovenia [67].

**Figure A2:**
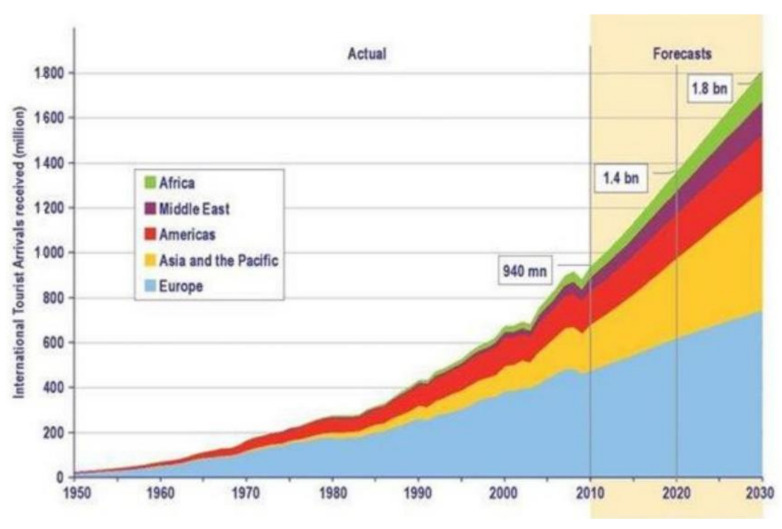
UNWTO prediction. Tourism towards 2030: Actual trend and forecast 1950–2030 [68].

## Data Availability

Not applicable.

## Appendix A

**Table A1:** The review of some most recent post-effect research of a pandemic.

Author(s)	Main Finding	Methodology
[57]	The main goal of this case study is to analyse the air traffic, air cargo, and the safety-hygiene air corridor between the United Kingdom (UK) and Spain. Air traffic that airlines project onto the UK-Spain corridor has decreased due to the pandemic. In the medium term, implementing the new Safety-Hygiene Air Corridor (SHAC) will return to nurture airlines, airports and destinations economically.	A case study
[58]	The drastic drop in flight frequencies at airports during the pandemic has caused an average decrease of 65% in passenger arrivals until October 2020, which is 23 million passengers; too many passengers for the Andalusian economy, which depends mainly on the tourism sector.	A review of the relevant literature, secondary data research
[59]	An instrument to measure the influence of coronavirus (COVID-19) on international travellers’ behaviour has been developed. Five hundred respondents in the Kingdom of Saudi Arabia were surveyed to create and validate a scale to measure international travel behaviour post-COVID-19. Findings revealed a hierarchical three-level scale for measuring international travellers’ behaviour. The tourism and hospitality industry can use the scale to assess the impact of COVID-19 or any future pandemic.	A questionnaire, Factor analysis
[60]	The COVID-19 virus was a cataclysmic event that will change the course of human history in numerous ways. At the time that this editorial was written, there were 113 million cases of infections worldwide, which resulted in 2.5 million deaths.	Expose
[62]	Econometric testing is conducted on a sample of 205 countries/territories. The results show a positive and significant relationship between COVID-19 and tourist arrivals per capita. This finding suggests that the tourism specialisation model in the small island context is too vulnerable to be considered sustainable in the medium to long term.	Time series
[63]	The monthly foodborne disease incidence in Shenzhen from January 2012 to December 2017 was between 954 and 32,863. The mean absolute percentage error (MAPE) was used to assess the model’s performance. The ARIMA (1,1,0) model was adequate for the change in month-to-month data.	ARIMA
[64]	Since 1980, the world has been threatened by different waves of emerging disease epidemics. It is difficult to stop the occurrence of new pathogens in the future due to the interconnection among humans, animals, and the environment. However, it is possible to face a new disease or reduce its spread risk.	Expose of time series
[65]	The paper provides a short-run estimation of international tourism demand focusing on the case of the FYR of Macedonia. The forecasted values of the chosen model can assist in mitigating any potential negative impacts on the country’s tourism development plan.	ARIMA

Source: authors’ compilations.

## Appendix B

**Table A2:** The robustness of the research, 156 observations, 2000 = 100 base indices, period 2008–2020.

Country	Results
Domestic tourists	Dummy variable, viruses of COVID-19, and Campylobacter are negatively significant.
Foreign tourists	A dummy variable (negatively) and *E. coli* (positively) are significant.
All inbound tourists	A dummy variable (negatively) and *E. coli* (positively) are significant.
Country-specific tourists (Germany)	A dummy variable and viruses of COVID-19 (negatively) and *E. coli* (positively) are significant.
Country-specific tourists (Netherlands)	A dummy variable (negatively) and *E. coli* (positively) are significant.
Country-specific tourists (Austria)	A dummy variable and viruses of COVID-19 are negatively significant.

Source: authors’ compilations based on NIPH [43] SORS [44] and SORSa [66] data. ARIMA (1,1,1).
